# Enhanced banana productivity, soil microbial community structure, and *Fusarium* wilt resistance by ultra-wide-narrow row planting pattern

**DOI:** 10.3389/fpls.2025.1753867

**Published:** 2026-02-25

**Authors:** Lina Huang, Shimin Cheng, Hailin Liu, Shaolong Sun, Rongxiang Wang, Zengxian Zhao, Junya Wei, Xiaolin Fan, Shouxing Wei

**Affiliations:** 1 Tropical Crops Genetic Resources Institute, Chinese Academy of Tropical Agricultural Sciences, Haikou, Hainan, China; 2 Key Laboratory of Crop Gene Resources and Germplasm Enhancement in Southern China, Ministry of Agriculture and Rural Affairs, Haikou, Hainan, China; 3 Key Laboratory of Tropical Crops Germplasm Resources Genetic Improvement and Innovation of Hainan Province/National Tropical Fruit Tree Variety Improvement Center, Haikou, Hainan, China; 4 Rubber Research Institute, Chinese Academy of Tropical Agricultural Sciences, Danzhou, Hainan, China; 5 College of Natural Resources and Environment, South China Agricultural University (SCAU), Guangzhou, Guangdong, China; 6 State Key Laboratory for Tropical Crop Breeding, Sanya, Hainan, China

**Keywords:** banana, *Fusarium* wilt mitigation, mechanized cultivation, microbial diversity, nutrient uptake, ultra-wide-narrow row planting pattern, yield

## Abstract

The sustainability of banana production is severely constrained by labor intensiveness, *Fusarium* wilt epidemics, and mechanization incompatibility under conventional planting systems. A three-year (2019-2021) investigation systematically evaluated three distinct planting configurations for simultaneously addressing these challenges while maintaining consistent plant density at 2220 plants ha^-^¹. The three planting configurations included T3M (3.0 m/1.5 m wide-narrow row, 2.0 m spacing), T5M (5.0 m/1.5 m ultra-wide-narrow row, 1.4 m spacing), T6M (5.0 m/1.5 m ultra-wide-narrow row, 1.2 m spacing). T5M demonstrated superior performance across agronomic and biochemical parameters. T5M outperformed T6M by 23.27-40.12% greater productivity with 8.13% higher fruit number per bunch, while maintaining T3M’s commercial harvest stability and fruit quality, stable and higher commercial harvest rates. T5M enhanced fruit quality over T6M in 2021, increasing total soluble solids by 15.81%, ascorbic acid by 18.35%, and sugar-acid ratio by 16.93%. T5M prioritized reproductive growth, increasing aboveground biomass 12.99% and fruit dry matter 18.73% over T3M in 2021.Compared to T6M, T5M improved nutrient cycling, enhanced aboveground N, P and K uptake by 15.68%, 12.88% and 12.26% respectively. Soil available nitrogen increased 18.19% versus T3M, with available potassium rosing 11.46% over T6M. T5M selectively modified soil microbiomes while preserving pH and organic matter stability. Microbial analysis showed Actinobacteriota enrichment increased 26.03% alongside *Fusarium* reduction by 31.50%, with redundancy analysis identifying organic matter as the major restructuring driver. These findings position the 5-meter ultra-wide-narrow row configuration as an innovative solution simultaneously enhancing yield, mechanization efficiency and pathogen suppression for sustainable banana production systems.

## 1 Introduction

As a globally pivotal tropical fruit, banana is cultivated across over 100 countries and regionswithin the latitudinal range of the Tropic of Capricorn and the Tropic of Cancer (Leonel et al., 2020; Price et al., 2020; Wang et al., 2025). In China, its agricultural and economic value is underscored by a cultivation area exceeding 340,000 hectares (Xie, 2019). However, sustainable banana production is increasing constrained by intertwined challenges requiring innovative agronomic solutions, particularly addressing labor-intensive management practices and persistent soil-borne disease pressures. Labor costs account for over 30% of total production inputs in Chinese, a burden further exacerbated by rural-to-urban workforce migration (Wang et al., 2016; Su et al., 2019; Guo et al., 2020). Concurrently, *Fusarium* wilt, caused by *Fusarium oxysporum* f. sp. *cubense* (Foc), inflicts devastating yield losses of 30-80% in infected plantations (Butler, 2013; Siamak and Zheng, 2018; Zeng et al., 2023; Zhang et al., 2024b). Conventional monoculture systems degrade soil microbial communities, while excessive chemical inputs exacerbate soil degradation and reduce nutrient use efficiency. These challenges collectively highlighted the need for systemic innovations in mechanization-compatible agroecosystems to balance banana productivity and ecological resilience.

Globally, advanced economies have integrated mechanization into pest control and precision irrigation (Li et al., 2011), while China’s banana industry lags in orchard-scale automation and non-destructive harvesting (Xu, 2019). A key bottleneck is conventional narrow row spacing (<3m), which restricts the maneuverability of large-scale machinery-a fundamental prerequisite for modern agroecological management (Huang et al., 2024). This underscores the urgency of redesigning planting architectures through spatio-temporal resource optimization. As an innovative agronomic practice, the wide–narrow row planting pattern multifunctional benefits in annual crops such as maize, cotton and wheat by optimizing spatial configuration (Lacolla et al., 2023; Tang et al., 2025; Xin et al., 2025). The studies on rice, wheat, and soybean have confirmed that wide-narrow row planting pattern significantly enhances yield and quality by improving light distribution, optimizing root architecture, increasing soil nutrient availability and boosting nutrient uptake efficiency (Hu et al., 2020; Xiong et al., 2022; Dong et al, 2023; Zhang et al., 2024a; Gao et al., 2025). Wider rows also enhance airflow, reducing humidity and the spread of fungal diseases, thereby minimizing pesticide application (Zhou et al., 2019; Zuo et al., 2023). Furthermore, this planting pattern offers significant advantages in labor savings and improving the efficiency of mechanized operations (Hu et al., 2021; Haarhoff and Swanepoel, 2022). Nevertheless, these gains have been primarily documented in short-stature annual crops. For perennial herbaceous crops such as banana, critical knowledge gaps remain, particularly regarding planting systems that reconcile mechanization with ecological function. Therefore, we propose an enhanced ultra-wide-narrow row planting pattern-a novel spatial configuration that strategically expands wide-row spacing for large-scale equipment passage while proportionally reducing narrow-row and inteplant spacing to maintain constant planting density. Despite its mechanization potential, the implementation of ultra-wide row configurations remains in banana cultivation underexplored. Most notably, the tripartite interplay between such configurations, soil microbiome resilience, and *Fusarium* wilt suppression-an essential relationship for sustainable banana production-has yet to be systematically investigated.

**Figure 1:**
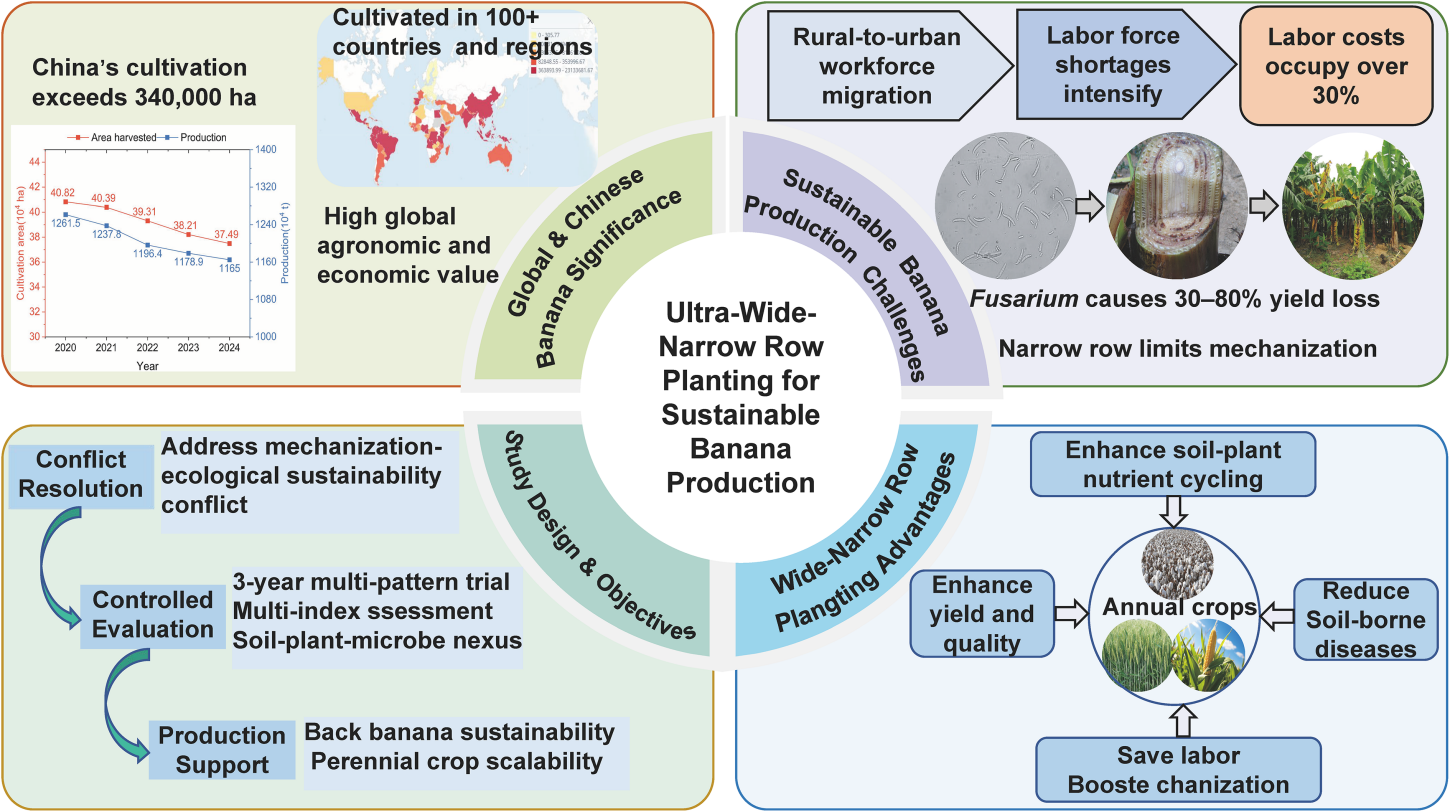
Research framework diagram of ultra-wide-narrow row planting pattern (Data quoted from China State Farms & Tropical Agriculture).

To bridge the gap between mechanization demands and ecological sustainability, this study implements the proposed ultra-wide-narrow row pattern enabling full-cycle mechanization (tillage, fertilization, harvesting) and systematically evaluates its performance. A three-year field experiment evaluated three such patterns under equal density, focusing on banana yield, nutrient dynamics, soil properties, and microbial diversity with simplified fertilization regimes. The findings are intended to provide theoretical and practical support for the development of efficient and sustainable banana production systems, as well as for the intensification of perennial banana cultivation (**Figure 1**).

## 2 Materials and methods

### 2.1 Experimental site and materials

The three-year field experiment (July 2019 to June 2021) was conducted in Hemian Village (19.825°N, 109.604°E), Lingao County, Hainan Province, China, a region characterized by a tropical monsoon climate with an mean annual temperature of 23°C (1991-2020) and precipitation averaging 1,418 mm without frost occurrence. The brick-red soil exhibited the following physicochemical properties pH 6.65, organic matter (OM) 43.70 g kg^-^¹, available nitrogen(AN) 76.37 mg kg*^-^*¹, available phosphorus (AK) 52.14 mg kg^-^¹, available potassium 145.85 mg kg^-^¹. The experimental material consisted of ‘*Formosana*’ wilt-resistant banana (*Musa acuminata L. AAA Cavendish.* cv. *‘Formosana’*) cultivated using standardized fertilization protocols. Nutrient inputs included compound fertilizer (15-15-15, N-P_2_O_5_-K_2_O), calcium superphosphate (16% P_2_O_5_), potassium chloride (60% K_2_O equivalent), and urea (46% N), with vegetable oil-coated controlled-release urea (44% N) constituting 50% of total nitrogen application.

### 2.2 Experimental design

**Figure 2:**
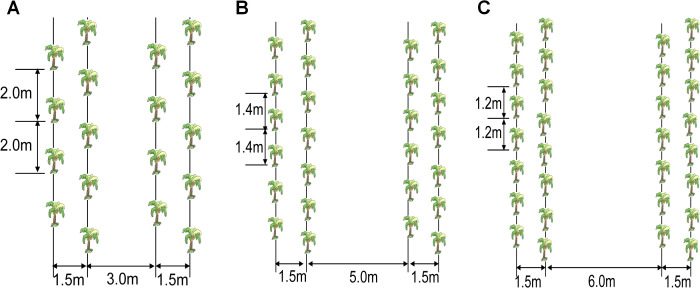
Schematic representation of the ultra-wide-narrow row planting patterns evaluated in the this study. **(A)** T3M, **(B)** T5M, **(C)** T6M.

A randomized completed block design with three replicates was employed to three planting configurations. The planting configurations evaluated were T3M conventional wide-narrow rows (3.0 m wide row, 1.5 m narrow row, 2.0 m interplant spacing), T5M ultra-wide-narrow rows (5.0 m wide row, 1.5 m narrow row, 1.4 m interplant spacing) and T6M ultra-wide-narrow rows (6.0 m wide row, 1.5 m narrow row, 1.2 m interplant spacing). All treatments maintained consistent planting density of 2,220 plants ha^-^¹ through optimized triangular spatial arrangements to maximize resource utilization efficiency (**Figure 2**).

Nutrient management followed standardized protocols uniformly applied to all experimental treatments. The total nutrient supply per hectare is 666 kg N, with total 226.4 kg P_2_O_5_, and 1287.6 kg K_2_O, establishing an N-P_2_O_5_-K_2_O ratio of 1-0.4-1.93. Fertilizers application involved band placement in 5-10 cm deep trenches maintained at 25 cm distance from the pseudostems, with immediate soil incorporation after application. Growth-stage-specific NPK allocation schedules and application timings are provided in **Table 1**. All plots received identical agronomic management throughout the study period.

**Table 1:** Fertilization schedule and NPK ratios of controlled-release blended fertilizer under simplified nutrient regimes.

Topdressing times	Growth stage	Application ratio(%)		
		N	P_2_O_5_	K_2_O
1^st^ time	Field seedling stage	15	20	5
2^nd^ time	Vigorous growth stage	30	30	20
3^rd^ time	Bud differentiation stage	30	25	30
4^th^ time	Flower bud development stage	20	20	30
5^th^ time	Fruit enlargement stage	5	5	15

### 2.3 Sampling and analytical methods

#### 2.3.1 Banana yield determination

At the inflorescence emergence stage, inflorescence emergence rate was calculated as the percentage of plants with visible inflorescence emergence relative to total planted individuals. Experimental evaluation ceased upon reaching the predefined phenological endpoint (80% flowering rate in T3M treatment), with triplicate sampling maintained throughout the observation period. At commercial maturity stage, three representative plants per experimental replicate were systematically selected for yield component quantification. Marketable bunch weight was measured using factory-calibrated digital scales. The marketable banana harvest rate was calculated as the percentage of harvested plants meeting commercial standards relative to the total planted individuals within each experimental replicate plot. For yield quantification purposes, the metric tons per hectare production value is derived through the sequential multiplication of individual plant yield expressed in kilograms, planting density per hectare, the marketable harvest rate percentage, followed by metric unit conversion scaling. This comprehensive assessment protocol enabled precise treatment effect evaluation on banana productivity (Xie et al., 2021).

#### 2.3.2 Fruit morphometric characterization​

Morphometric analysis targeted the third fruit hand from sampled plants to ensure developmental consistency. From each selected hand, six fingers (three upper and three lower layer fruits) underwent dimensional analysis to minimize positional bias. Researchers measured fruit curvature length (pedicel excluded) using flexible measuring tapes and determined maximum circumference at the equatorial region. The fruit index calculation incorporated both numerical counts per bunch and individual finger mass (± 0.01 g precision) obtained through analytical weighing g (Huang et al., 2017b).

#### 2.3.3 Fruit quality evaluation

Fruits from the third cluster of each plant were subjected to artificial ripening prior to quality assessment. Three fruits per plant were sampled with nine biological replicates per treatment. Total soluble sugars (TSS) were quantified by the anthrone-sulfuric acid method while Soluble solids content (SSC) was determined using a digital refractomete (Tian et al., 2024). Titratable acidity (TA) was measured through standardized neutralization titration and ascorbic acid (AsA) content was analyzed via 2,6-dichlorophenolindophenol titration following established protocols (Anchana et al., 2025; Xu et al., 2020). Technical triplicates were performed for all measurements with appropriate quality controls.

#### 2.3.4 Plant biomass partitioning and nutrient analysis

Whole (Aboveground) plant dissection separated stem (including pseudostems and true stems), leaf components, and fruit tissues from three representatives per replicate. Following longitudinal quartering of stem tissues and petiole-amina separation, these subsamples were homogenized through standardized coning-and-quartering procedures (Huang et al., 2017a). Fresh weights (± 0.01 g resolution) preceded constant-mass drying at 75°C (± 2°C) for dry matter determination. Milled tissues were digested with H_2_SO_4_-H_2_O_2_, followed by colorimetric and spectrometric analyses: total nitrogen (TN) via alkaline distillation, total phosphorus (TP) via molybdenum–antimony colorimetry, and total potassium (TK) via flame photometry (Lu, 2000).

#### 2.3.5 Soil physicochemical assessment

Post-harvest soil sampling procedures conducted in 2021 to evaluate how ultra-wide-narrow row planting influences soil properties and microbial communities in banana plantations. Composite samples (0-20 cm depth) were systematically collected from fifteen randomly chosen banana plants per treatment replicate, with sampling confined to a 50 cm radius surrounding each pseudostem base following removal of surface debris. After field homogenization and debris removal, representative 1 kg soil samples were immediately transferred into pre-sterilized polyethylene bags maintained in dry ice (-80°C) boxes during transport to the laboratory within 24 h to minimize microbial activity and physicochemical alterations. Sample processing involved splitting aliquots for separate physicochemical and microbial analyses. one fraction underwent controlled air-drying (<30°C) and sieving (18-mesh, 1 mm) for determination of fundamental soil characteristics including pH (water:soil ratio 5:1) via potentiometry, OM via potassium dichromate oxidation, AN via alkaline diffusion, AP via molybdenum-antimony colorimetry, and AK via flame photometry (Yang et al., 2020).

#### 2.3.6 Soil microbial community profiling​

Molecular analysis utilized cryopreserved (-80°C) soil aliquots from physicochemical sampling. Total DNA was extracted using the E.Z.N.A.^®^ DNA Kit, with purity and concentration verified via 2% agarose gel electrophoresis. The bacterial 16S rRNA V4-V5 region was amplified using primers 338F (5’-ACTCCTACGGGGAGGCAGCAG-3’) and 806R (5’-GGACTACHVGGGGTWTCTAAT-3’), while fungal ITS1-ITS2 regions were amplified with primers ITS1F (5’-CTTGGTCATTTAGAGGAAGTAA-3’) and ITS2R (5’-GCTGCGTTCTTCATCGATGC-3’). Amplicons were pooled, gel-purified using the AxyPrep DNA Gel Recovery Kit, and sequenced on an Illumina MiSeq PE300 platform (Majorbio Co.) to characterize microbial community structure (Zhang et al., 2025).

### 2.4 Statistical processing

Raw sequencing data were processed through splicing, quality control, and filtering to remove chimeras, yielding optimized sequences; those with ≥97% similarity were clustered into operational taxonomic units (OTUs). Microbial α-diversity indices (Ace, Chao1, Shannon, Simpson) were calculated in QIIME 1.9.1 to assess community diversity and richness Community composition variability across taxonomic levels was analyzed in R 3.3.1 (Barberan et al., 2012). Agronomic data were analyzed via one-way ANOVA with Duncan’s *post-hoc* test in SPSS 25 to determine treatment differences, and visualizations were produced in Origin 2022 and R for clear result interpretation.

## 3 Results

### 3.1 Banana productivity

**Figure 3:**
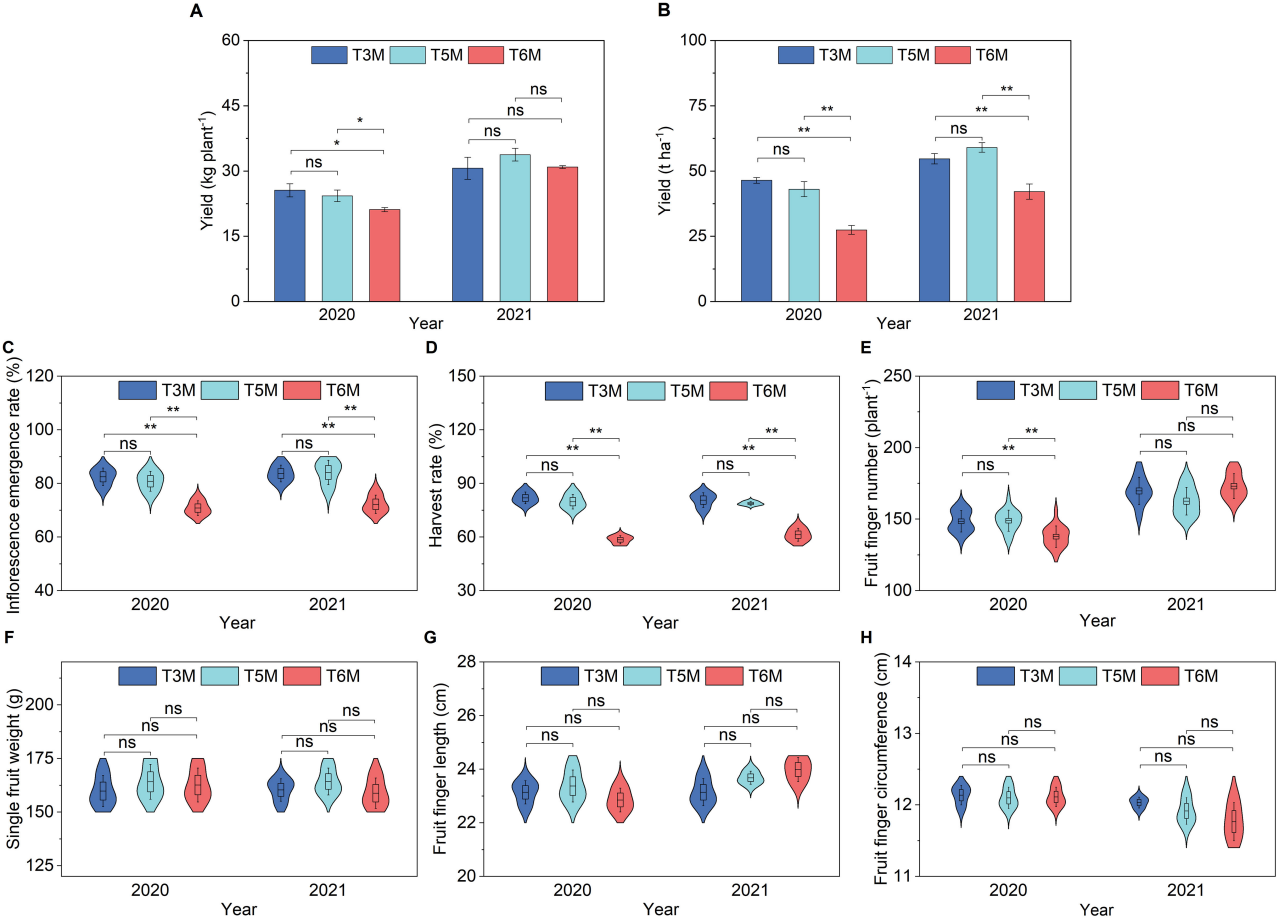
The yield and its components of banana under wide-narrow row planting patterns. **(A)** Yield per plant, **(B)** Total yield, **(C)** Inflorescence emergence rate, **(D)** Harvest rate, **(E)** Fruit finger number, **(F)** Single fruit weight, **(G)** Fruit finger length, **(H)** Fruit finger circumference. All the values are representative of three independent experiments. Significance of Duncan ‘s new multiple range test: ns, not significant (*P* ≥ 0.05); *P < 0.05, **P < 0.01. Same as the figures below.

The ultra-wide-narrow row planting patterns demonstrated different effects on banana productivity across two consecutive cultivation cycles (2019-2020 and 2020-2021). Initial observations revealed equivalent marketable yields per plant between T5M and T3M in 2020, indicating minimal yield penalty from moderate spacing adjustments. Notably, both T5M and T3M outperformed T6M by 15.00% and 21.01% respectively in 2020, indicative of fundamental limitations in resource capture efficiency inherent to excessively spaced planting arrangements. By 2021, yield variations across treatments diminished significantly, with plant-level productivity converging within a narrow range (30.63-33.75 kg) and showing no statistical separation based on one-way ANOVA (**Figure 3A**). This temporal homogenization implies physiological adaptation mechanisms involving root architecture plasticity and canopy light-use optimization under sustained spacing conditions. Multi-annual analysis positioned T5M as maintaining consistent hectare-scale yield parity with T3M while sustaining 40.25-56.81% productivity advantages over T6M (**Figure 3B**). These findings position T5M as representing an optimal balance between spatial resource allocation and photosynthetic efficiency, where diminishing returns in yield potential become biologically unavoidable beyond certain spatial thresholds.

The ultra-wide-narrow row planting patterns induced multidimensional optimization of banana yield components with significant restructuring effects (**Figures 3C–H**). T5M achieved inflorescence emergence rates, marketable banana harvest rates comparable to T3M while exhibiting minimal interannual variability (<5% fluctuation between 2020-2021), demonstrating the robustness of moderate row width modifications in maintaining commercial productivity. Conversely, T6M manifested substantial yield penalties, reducing inflorescence emergence rate by 10.00%-11.83% reduction and commercial harvest rate by 17.52%-23.46% reduction across two cycles (**Figures 3C, D**). Detailed fruit morphogenesis analysis revealed that T5M and T3M yielded 8.13% and 7.84% more total finger numbers per bunch than T6M respectively in 2020, reinforcing the superior performance of T5M (**Figure 3E**). Crucially, no treatment-induced variations occurred in individual fruit morphometric parameters (weight, length, or circumference), indicating spacing effects primarily mediated yield through fruit quantity (**Figures 3F–H**). Hierarchical productivity gains positioned T5M as achieved T3M-equivalent bunch weights while delivering 15.00% (2020) to 40.25-56.81% (multi-annual) yield enhancements over T6M at hectare scale. This superior productivity hierarchy cements T5M as an agronomic benchmark, where physiological resource partitioning aligns optimally with canopy architecture to maximize reproductive efficiency. Such synchronization ensures both yield stability and operational efficiency through reduced harvest labor intensity. Such alignment of productivity enhancement with ecological intensification principles presents a paradigm for sustainable banana cultivation systems.

### 3.2 Banana quality

**Figure 4:**
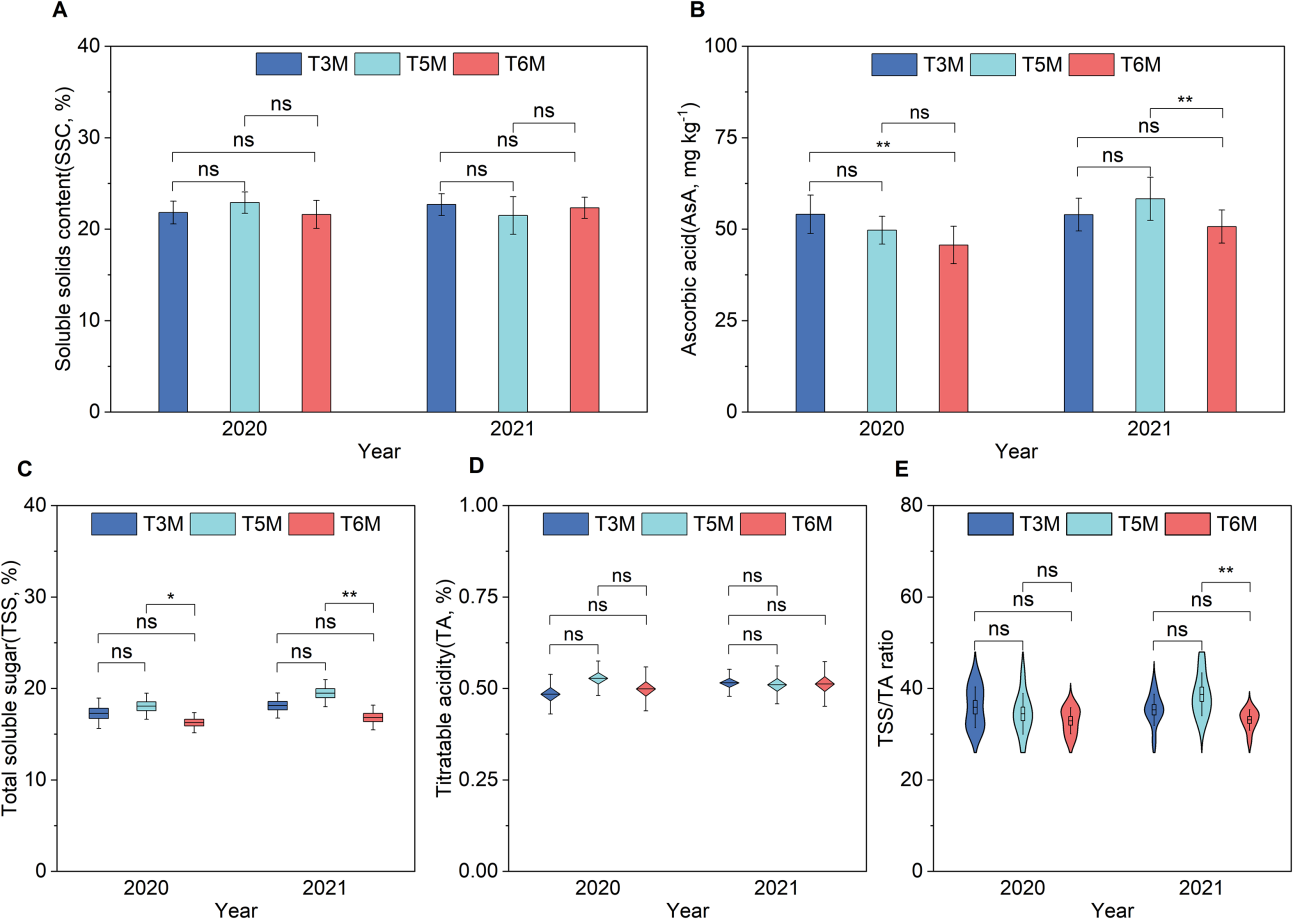
The quality of banana under wide-narrow row planting patterns. **(A)** SSC, **(B)** AsA, **(C)** TSS, **(D)** TA, **(E)** TSS/TA ratio. Significance of Duncan’s new multiple range test: ns, not significant (P ≥ 0.05); *P < 0.05, **P < 0.01.

The effects of row spacing configurations demonstrated clear temporally distinct impacts on banana fruit quality. SSC and TA remained stable across three spacing treatments in both years (**Figures 4A, D**). AsA accumulation displayed pronounced seasonal variation. Compared to T6M, T3M exhibited maximal elevation by 18.35% in 2020 while T5M showed strongest performance with an 14.99% increase in 2021 (**Figure 4B**). TSS showed progressive enhancement under ultra-wide-narrow spacing, with T5M demonstrating consistent superiority, achieving 11.00% and 15.81% increases relative to T6M in 2020 and 2021 respectively (**Figure 4C**). The sugar-acid ratio followed similar temporal patterns to TSS, with T5M improving by 16.93% compared to T6M in 2021 (**Figure 4E**). The 5-meter ultra-wide-narrow configuration achieved parity with conventional spacing in overall quality while progressively amplifying the accumulation of both primary (TSS) and secondary (AsA) metabolites across consecutive seasons. These findings underscore the metabolic plasticity of banana under optimized planting geometries, suggesting that strategic row spacing can fine-tune sink-source relationships to enhance nutritive and flavor compounds without compromising yield stability.

### 3.3 Banana dry matter allocation and partitioning

**Figure 5:**
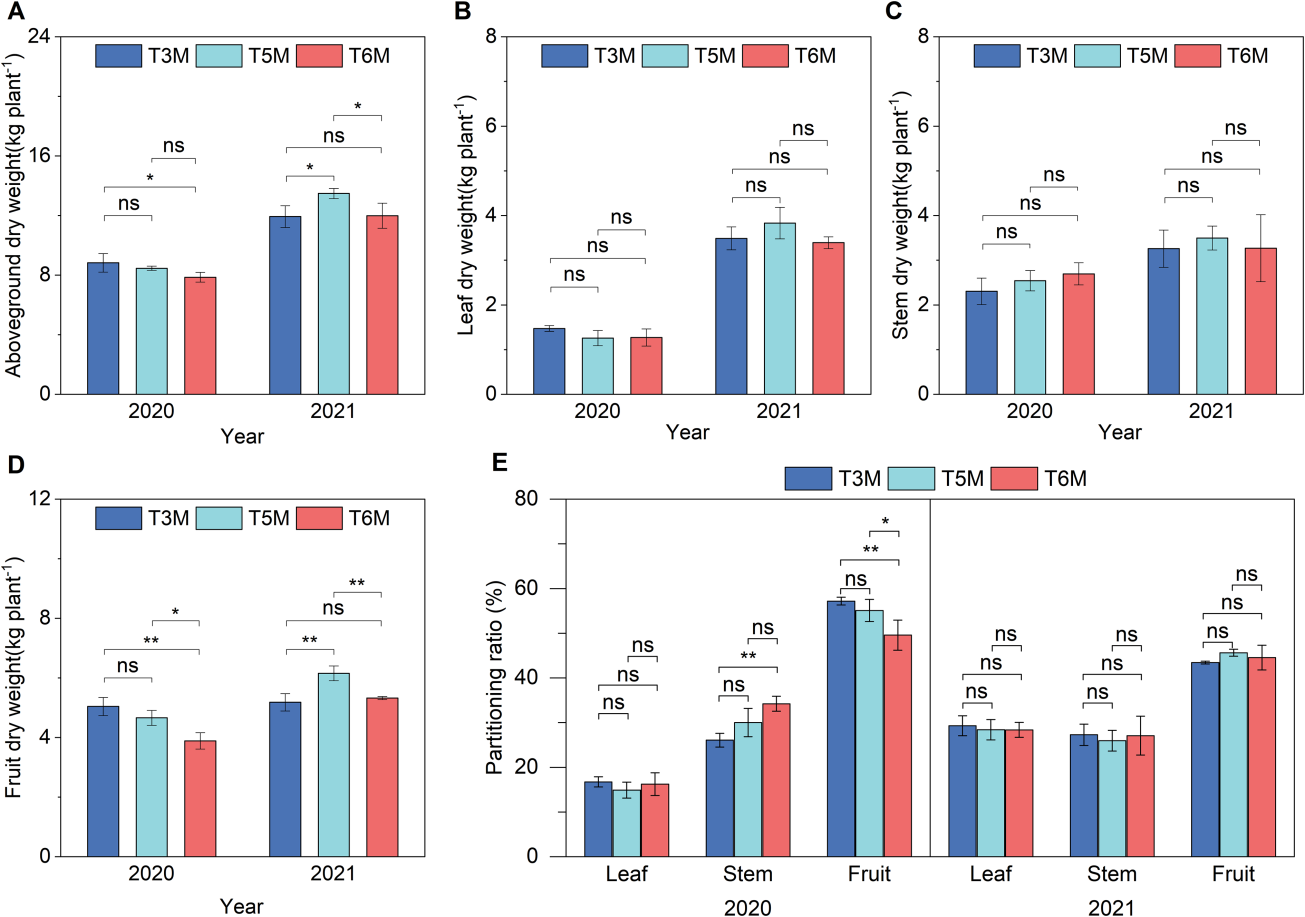
Banana dry matter accumulation and partitioning under wide-narrow row planting patterns. **(A)** Aboveground dry weight, **(B)** Leaf dry weight, **(C)** Stem dry weight, **(D)** Fruit dry weight, **(E)** Partitioning ratio. Significance of Duncan’s new multiple range test: ns, not significant (P ≥ 0.05); *P < 0.05, **P < 0.01.

The implementation of ultra-wide-narrow row configurations significantly altered dry matter partitioning dynamics in banana plants across consecutive growing seasons (**Figure 5**). Initial evaluation in 2020 showed aboveground dry weight of T5M was comparable to that of T3M and T6M, whereas T3M significantly outperformed T6M by 12.50%. This balance shifted markedly in subsequent season, with T5M emerging as the dominant configuration through its significant biomass accumulation (13.47 kg plant^-^¹) that surpassed T3M and T6M by 12.91% and 12.34% respectively, confirming T5M’s capacity for sustained productivity enhancement (**Figure 5A**). The most striking treatment effects emerged in reproductive dry matter allocation patterns. T5M consistently maximized fruit dry matter production-initially outperforming T6M by 19.79% (2020) before extending its advantage to 15.60% over T6M and 18.73% over T3M (2021) (**Figure 5D**). Further analysis of dry matter partitioning ratios among aboveground components revealed that T3M and T5M significantly increased the proportion of fruit dry matter by 15.37% and 11.14% respectively relative to T6M in 2020 (**Figure 5E**). The contrasting stability observed in vegetative organ partitioning suggested fruit development preferentially assimilates benefits from spatial optimization (**Figures 5B, C, E**). Such hierarchical resource allocation likely stems from the T5M’s configuration balancing the distribution of photosynthetically active radiation and the accessibility of root-zone nutrients, whereas T6M’s configuration disproportionately affected reproductive growth.

### 3.4 Banana nutrient accumulation and partitioning

**Figure 6:**
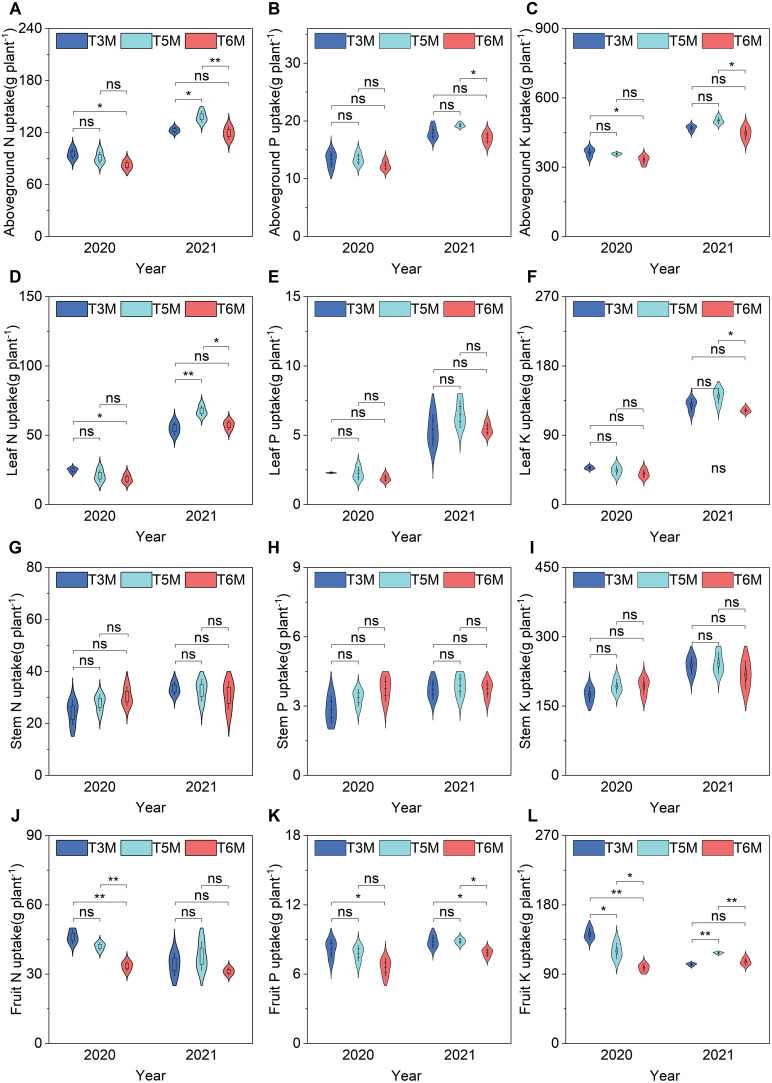
Banana nutrient accumulation and partitioning under wide–narrow row planting patterns. **(A)** Aboveground N uptake, **(B)** Aboveground P uptake, **(C)** Aboveground K uptake, **(D)** Leaf N uptake, **(E)** Leaf P uptake, **(F)** Leaf K uptake, **(G)** Stem N uptake, **(H)** Stem P uptake, **(I)** Stem K uptake, **(J)** Fruit N uptake, **(K)** Fruit P uptake, **(L)** Fruit K uptake. Significance of Duncan’s new multiple range test: ns, not significant (P ≥ 0.05); *P < 0.05, **P < 0.01.

The ultra-wide-narrow row planting pattern significantly reorganized banana nutrient allocation strategies, exhibiting distinct temporal and organ-specific dynamics (**Figure 6**). Progressive optimization of aboveground macronutrient acquisition was most pronounced under T5M spacing. In 2020, 3M showed initial superiority with 15.61% N and 9.84% K enhancements compared to T6M (**Figure 6A**). T5M demonstrated superior optimization of aboveground nutrient capture in 2021, significantly enhancing N, P, and K uptake by 15.68% (**Figure 6A**), 12.88% (**Figure 6B**), and 12.26% (**Figure 6C**) respectively compared to T6M. Notably, comparative analysis between different planting configurations revealed T5M’s particular advantage in N assimilation over T3M, exhibiting a 12.58% increase in aboveground N uptake (**Figure 6A**). This progressive nutrient acquisition pattern underscores T5M’s effectiveness in orchestrating balanced macronutrient uptake under ultra-wide-narrow row planting systems.

Leaf tissues exhibited selective nutrient sensitivity, forming the primary sink for spatial adjustments with N being particularly responsive. In 2020, T3M demonstrated superior performance with a 15.61% increase in aboveground N uptake compared to T6M, whereas T5M exhibited transitional equivalence to T6M. By 2021, under T5M spacing, leaf N uptake consistently improved by 17.91%-22.75% (**Figure 6D**), while potassium accumulation exhibited a moderate yet statistically significant increase of 15.37% relative to T6M (**Figure 6F**). Stem tissues maintained consistent nutrient profiles across treatments, confirming their limited role in spatial adaptation mechanisms (**Figures 6G–I**).

Fruit nutrient deposition demonstrated progressive optimization with modified geometry. In 2020, T3M increased N, P, and K uptake by 44.66%, 24.09%, and 44.66% respectively versus T6M in 2020, while T5M showed 22.25% and 22.34% improvements in N and K uptake. In 2021, the P and K uptake of T5M increased by 12.60% and 10.54% respectively compared with T6M, with the K uptake showing a particularly significant 14.71% elevation over T3M (**Figures 6J–L**). The results demonstrated that the ultra-wide-narrow row planting system induced systematic modifications in banana nutrient partitioning, differentially regulating N optimization in leaves, stable nutrient profiles in stems, and coordinated N-P-K accumulation in fruits, revealing distinct organ-specific adaptation strategies. Importantly, T5M emerged as the most effective treatment and maximized overall nutrient acquisition while maintaining balanced partitioning among aerial organs.

### 3.5 Soil agrochemical properties

**Figure 7:**
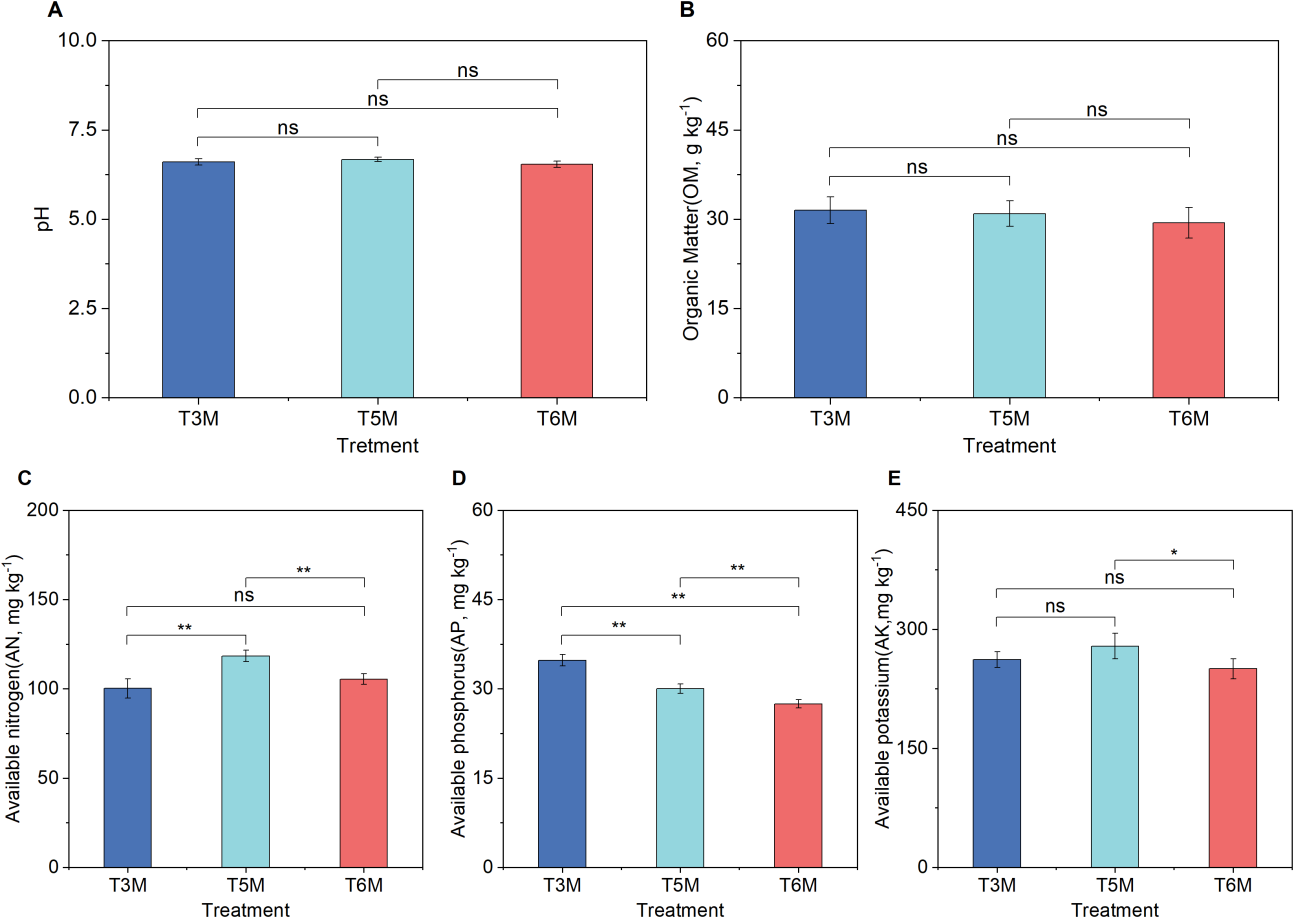
Soil agrochemical properties under wide-narrow row planting patterns. **(A)** pH, **(B)** OM, **(C)** AN, **(D)** AP, **(E)** AK. Significance of Duncan’s new multiple range test: ns, not significant (P ≥ 0.05); *P < 0.05, **P < 0.01.

The ultra-wide-narrow row planting patterns showed no significant impact on soil pH (6.54-6.67, **Figure 7A**) or organic matter content (2.94%-3.15%, **Figure 7B**) after two consecutive growing cycles, indicating stability in these fundamental soil parameters. However, distinct configurations elicited notable shifts in soil nutrient availability. The T5M regime significantly AN levels, exceeding T3M by 18.19% and T6M by 12.33% (**Figure 7C**), while sustaining superior AK (11.46% higher than T6M, **Figure 7E**). In contrast, T3M optimized phosphorus availability, surpassing T5M and T6M by 15.93% and 26.65%, respectively (**Figure 7D**). These divergent responses suggest that row spacing modifies nutrient cycling mechanisms-potentially mediated by microbial activity or root exudate profiles-without altering basic soil chemistry. Such selective nutrient modulation highlights the importance of spatial planting design in sustaining soil fertility and microbial-driven processes in intensive banana production systems.

### 3.6 Soil microbial diversity

**Figure 8:**
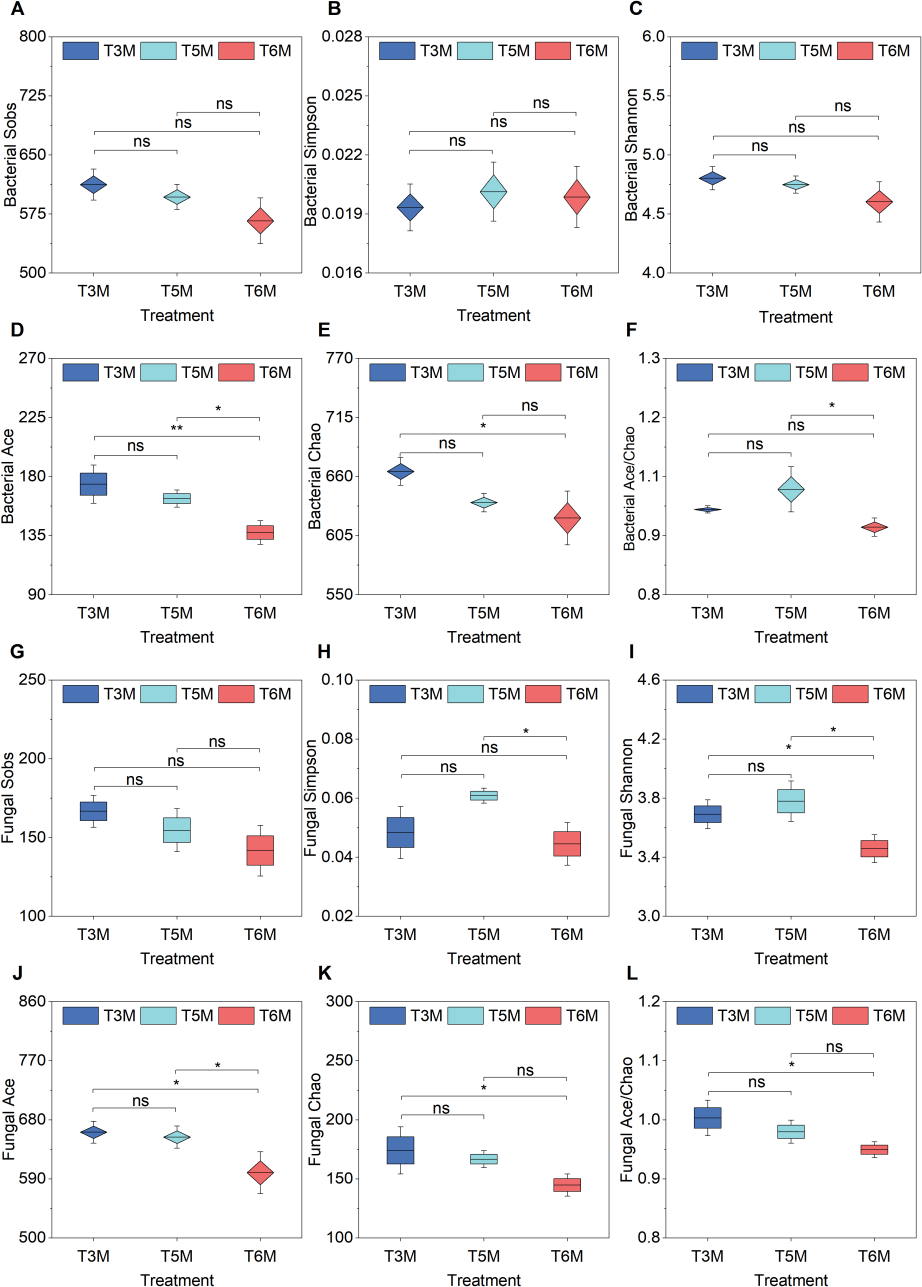
Soil microbial diversity under wide-narrow row planting patterns. **(A)**Bacterial Sobs, **(B)** Bacterial Simpson, **(C)** Bacterial Shannon, **(D)** Bacterial Ace, **(E)** Bacterial Chao, **(F)** Bacterial Ace/Chao, **(G)** Fungal Sobs, **(H)** Fungal Simpson, **(I)** Fungal Shannon, **(J)** Fungal Ace, **(K)** Fungal Chao, **(L)** Fungal Ace/Chao. Significance of Duncan’s new multiple range test: ns, not significant (P ≥ 0.05); *P < 0.05, **P < 0.01.

High-throughput sequencing generated 442,519 high-quality bacterial sequences and 491,026 high-quality fungal sequences, with genus-level alpha diversity patterns distinctly illustrated across row spacing configurations (**Figure 8**). With sample coverage indices universally reaching 1.00 across all treatments, microbial community assessments demonstrated reliability. Bacterial communities demonstrated remarkable stability regardless of row spacing variations (**Figures 8A–C**), though notable treatment-specific differences emerged in species richness estimators (Ace, Chao, Ace/Chao indices) (**Figures 8D–F**). Specifically, T3M exhibited respective 10.27% and 6.97% elevations in Ace and Chao indices compared to T6M, while T5M showed a 9.03% higher Ace index than T6M (**Figures 8D, E**). Importantly, the composite Ace/Chao index for T5M surpassed T6M by 7.29% (**Figure 8F**), reinforcing its capacity to sustain microbial richness. No statistically significant differences existed between T3M and T5M regarding Simpson or Ace indices, suggesting that T3M and T5M similarly preserved bacterial diversity. In striking contrast, fungal communities displayed pronounced sensitivity to spatial arrangements, diverging from the bacterial response patterns (**Figures 8G–L**). Specifically, T5M significantly increased fungal Simpson index by 13.51% and Shannon diversity by 9.25% relative to conventional T6M, whereas T3M demonstrated intermediate performance without statistically distinguishable differences from either extreme configuration (**Figures 8H, I**). Regarding species richness metrics, T3M surpassed T6M by 6.65% (Shannon), 19.88% (Ace), 20.29% (Chao), and 5.69% (Ace/Chao) (**Figures 8I–L**), while maintaining comparable Ace/Chao indices to T5M (**Figure 8L**).

Our findings demonstrate that planting system design exerts stronger regulatory effects on fungal communities than on bacterial assemblages, reinforcing fungi as sensitive bioindicators of planting spatial configuration. The T5M system emerges as the superior configuration, uniquely combining robust bacterial community stability with enhanced fungal diversity-a dual advantage critical for maintaining belowground ecological balance while supporting sustainable banana production.

**Figure 9:**
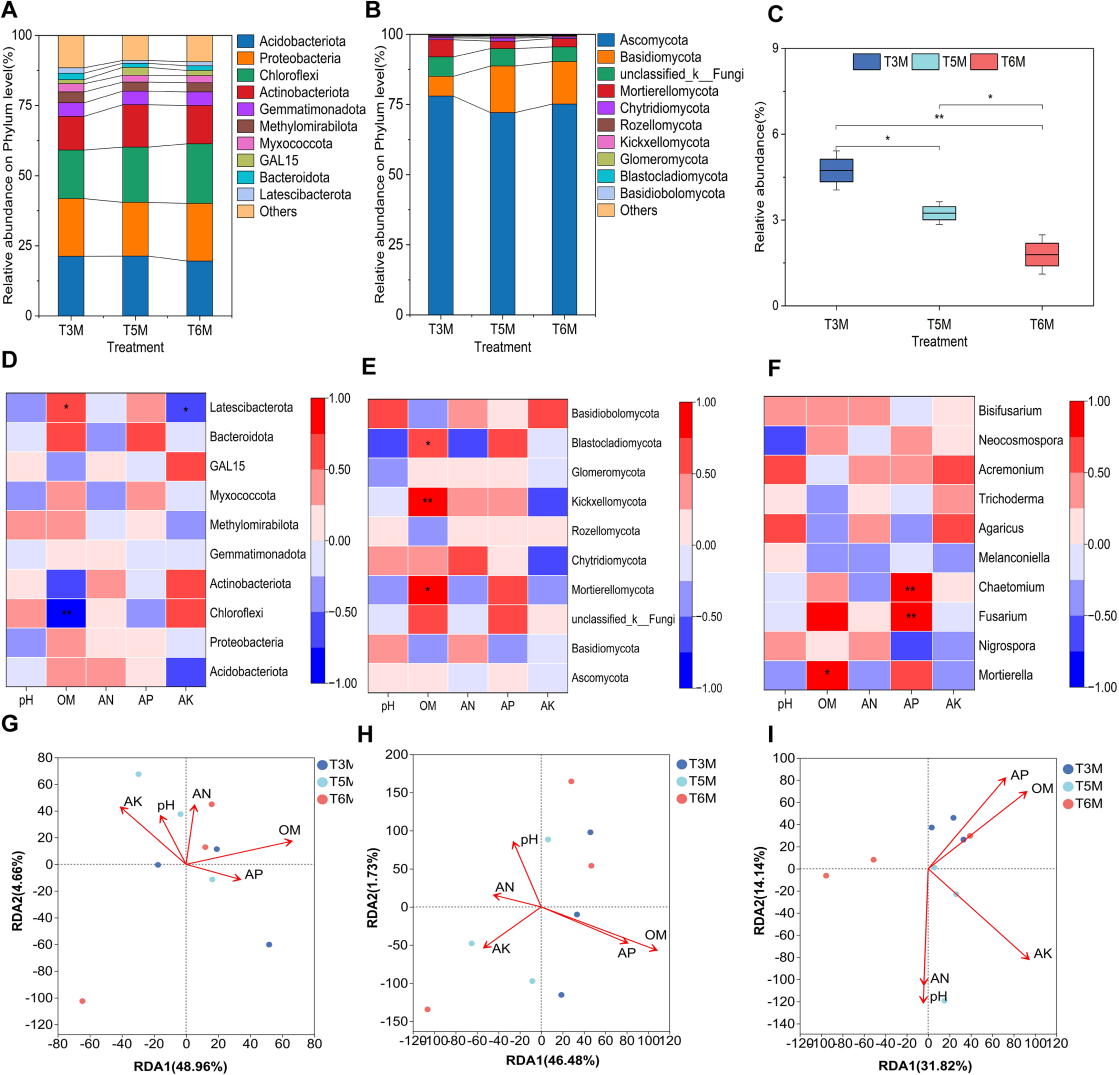
Microbial community dynamics in banana soils under ultra-wide-narrow row planting patterns. **(A, B)** Taxonomic shifts in bacterial **(A)** and fungal **(B)** phylum. **(C)** Relative abundance of *Fusarium* spp. **(D-F)** Spearman correlation heatmaps depicting bacterial **(D)** and fungal **(E, F)** interactions on phylum and genus levels. **(G-I)** Redundancy analysis (RDA) illustrating environmental influences on bacterial **(G)** and fungal **(H, I)** communities. Environmental factors are represented by arrows (length=explanatory strength; angle= correlation direction). Significant differences are marked (*P < 0.05, **P < 0.01).

Phylogenetic analyses indicated that four bacterial phyla (Acidobacteriota, Proteobacteria, Chloroflex and Actinobacteriota) persistently dominated the prokaryotic communities across all three wide-narrow row planting patterns, with their relative abundances averaging 73.79% of the total prokaryotic community (**Figure 9A**). Quantitative assessments demonstrated mean relative abundances of 20.70%, 20.10%, 19.45% and 13.54% for these phylum respectively. Ultra-wide-narrow row configurations elicited distinct modulatory effects on microbial composition. Acidobacteriota abundances in T3M (21.25%) and T5M (21.30%) showed 8.75% and 9.01% increases compared to T6M (19.52%), with elevated levels correlating with enhanced recalcitrant carbon utilization capacity that facilitates soil organic carbon accumulation and sequestration as documented in previous studies (Kielak et al., 2016; Kang et al., 2021; Zheng et al., 2022). Comparative analysis revealed Proteobacteria maintained a 7.14% higher abundance in T6M (20.57%) versus T5M (19.18%), while Chloroflexi demonstrated 22.44% and 13.35% dominance increases in T6M (21.28%) and T5M (19.70%) relative to T3M. Actinobacteriota in T5M (15.06%) and T6M (13.62%) exceeded T3M by 26.03% and 13.97% respectively, with this enrichment showing significant positive correlations with chitinase activity that promotes nitrogen mineralization while concurrently inhibiting Fusarium pathogen proliferation, thereby optimizing soil nitrogen cycling dynamics and plant health parameters as supported by multiple studies (Hui et al., 2020; Djemouai et al., 2023; Lee et al., 2023). Although planting configurations did not fundamentally alter dominant taxonomic composition, they significantly modulated relative abundances to establish optimized microbial functional profiles conducive to sustainable banana cultivation and disease suppression, particularly noting T5M’s enhancement of nitrogen mobilization through Actinobacteriota activation.

Fungal community assessments (**Figure 9B**) identified Ascomycota (75.08%) and Basidiomycota (12.91%) as the consistently dominant phyla across all planting patterns, with substantially lower representation from subordinate taxa including unclassified_k_Fungi (5.25%-6.97%) and Mortierellomycota (2.67%-6.08%), while Chytridiomycota and Rozellomycota remained below detection thresholds. Treatment-specific analyses showed Ascomycota maintained phylum-level dominance across all configurations (T3M 77.99%, T5M 72.12%, T6M 75.14%), whereas Basidiomycota exhibited 136.04% and 115.81% abundance increases in T5M (16.57%) and T6M (15.15%) versus T3M. Conversely, Mortierellomycota displayed significant 56.09% and 50.99% depletion in T5M and T6M respectively compared to T3M, clearly demonstrating the configuration-specific regulatory capacity of ultra-wide-narrow row planting systems on fungal community architecture on phylum level. These systematic investigations confirm that 5m/6m ultra-wide-narrow row planting patterns preserve fundamental fungal phylum composition while strategically rebalancing dominant taxa proportions. Genus-level resolution analysis identified *Fusarium*-the etiological agent of banana wilt disease-among the top 10 dominant fungal genera across all treatments (mean relative abundance 3.25%), with ultra-wide-narrow row planting patterns exerting differential suppression effects whereby T5M and T6M achieved 31.50% and 62.16% reductions respectively compared to T3M planting (**Figure 9C**). The pronounced *Fusarium* suppression under modified planting geometries, particularly the marked reduction in T6M, substantiates enhanced biological control potential against banana wilt disease, providing mechanistic insights for developing integrated cropping systems that simultaneously enhance productivity and pathogen suppression capacity.

Spearman rank correlation analysis delineated intricate linkages between environmental factors and soil microbial taxa, with correlation coefficients and statistical significance quantifying these biological relationships (**Figures 9D–F**). The analysis uncovered divergent nutrient-response patterns among bacterial phylum, fungal phylum and fungal genus. Latescibacterota demonstrated strong positive correlations with organic matter (OM), suggesting OM-dependent syntrophic interactions whereby this taxa presumably utilize OM decomposition products as metabolic substrates. Conversely, Chloroflexi showed pronounced negative correlations with OM, indicative of specialized adaptations to nutrient–poor environments where oligotrophic conditions favor taxa with superior nutrient acquisition strategies. Notably, Latescibacterota exhibited negative correlations with available potassium (AK), revealing taxon-specific nutrient sensitivities (**Figure 9D**). Fungal phylum analysis identified Mortierellomycota and Blastocladiomycota as OM-responsive biomarkers, with Kickxellomycota displaying particular OM affinity, collectively underscoring OM’s pivotal role in shaping fungal community assemblages (**Figure 9E**). Particularly noteworthy was *Fusarium*’s positive association with AP-a mechanistic linkage attributable to its phosphatase-mediated mineralization of organic phosphorus, consequently elevating bioavailable phosphorus pools within the rhizosphere (**Figure 9F**). These findings illuminate nutrient-mediated microbial niche partitioning within agroecosystems.

Redundancy analysis (RDA) revealed distinct environmental drivers structuring microbial communities under the ultra-wide-narrow row planting system (**Figures 9G–I**). Bacterial phylum assemblages showed pronounced hierarchical structuring along constrained RDA axes, which collectively explained 53.62% of total variance. Organic matter (OM) emerged as the predominant control over AK, AP, AN and pH parameters. This OM-driven pattern reflected its dual role as both carbon substrate and mediator of physicochemical conditions governing bacterial niche partitioning (**Figure 9G**). Fungal communities exhibited analogous structuring, with phylum–level variation primarily regulated by OM (46.47% variance explained), followed by AP, AK, pH and AN (**Figure 9H**). Genus-level fungal assemblages displayed more balanced environmental determinism (RDA1: 31.82%; RDA2; 14.14%), suggesting complex multidimensional niche filtering beyond simple hierarchy. Comparative analysis indicates this cropping system fundamentally restructures rhizosphere microbiomes through OM-mediated mechanisms that concomitantly reduce *Fusarium* prevalence. The emergent bio-barrier effect likely involves root exudate-induced shifts in quorum sensing networks, though precise metabolic pathways controlling this microbial reprogramming require longitudinal metabolome-proteome analyses.

## 4 Discussion

### 4.1 Yield optimization and quality maintaining of banana through ultra-wide-narrow row planting

Banana yield components, quality and soil microbial diversity underpin fruit production and cultivation profitability, serving as core metrics to evaluate the efficacy of ultra-wide-narrow row planting pattern. Despite rising demand for mechanized banana production, research on such spatial configurations remains limited (Huang et al., 2024). The ultra-wide-narrow row planting pattern redefines banana cultivation dynamics by simultaneously addressing yield stability and fruit quality considerations. Empirical evidence from 2019-2021 production cycles demonstrates that the 5 m spacing configuration (T5M) achieves critical equilibrium between productivity preservation (30.63-33.75 kg/plant, **Figure 3A**) and hectare-scale yield advantages (40.25-56.81%, **Figure 3B**) over wider spacing (T6M), while maintaining parity with conventional 3 m spacing (T3M). Yield component analysis revealed T5M maintained inflorescence emergence rates and commercial harvest rates equivalent to T3M (<5% interannual variability) while substantially outperforming T6M by 10.00-11.83% and 17.52-23.46% reductions respectively (**Figures 3C, D**), with fruit morphogenesis showing T5M produced 8.13% more fingers per bunch than T6M without affecting individual fruit weight, length or circumference (**Figures 3F–H**). Quality assessments showed T5M simultaneously achieved quality parameter parity with T3M (**Figure 4**) while surpassing T6M in key nutritive compounds. T5M progressively increased TSS by 15.81% and AsA by 14.99% relative to T6M by 2021, while maintaining sugar-acid ratio improvements of 16.93% (**Figures 4B, C**). This aligns with mechanized orchard studies where intermediate row widths (3-5m) maximized fruit-bearing site utilization without compromising mechanization compatibility (Qu et al., 2024). The hectare-scale yield and quality advantage of T5M over T6M, aligning with field crop studies where optimized wide-narrow rows enhance productivity (Yang, 2024; Wang et al., 2015; Liu et al., 2022; Wang et al., 2022, Wang et al., 2024), underscores the existence of a critical spatial threshold beyond 5.0 m where disproportionate resource allocation detrimentally affects reproductive investment-a finding with profound implications for precision planting design in *Musaceae* systems. These results collectively establish ultra-wide-narrow row planting pattern (T5M) as an aninnovative, high-performance alternative to conventional banana cultivation approaches, potentially transforming production paradigms through simultaneous yield enhancement and mechanization compatibility.

### 4.2 Dry matter accumulation and nutrient partitioning dynamics

The ultra-wide-narrow row planting system exerted significant influence over dry matter accumulation patterns and nutrient partitioning strategies in banana plants. Spatial configurations imposed distinct hierarchies in dry matter distribution and nutrient allocation strategies. While initial-season biomass accumulation showed treatment parity (2020), T5M developed pronounced superiority by 2021 with 12.43%-12.99% greater aboveground dry weight than comparators (**Figure 5A**)-a delayed response implicating carbohydrate reserve mobilization mechanisms. The configuration’s preferential enhancement of fruit dry matter (19.79% and 15.60% over T6M) without altering vegetative organ partitioning (**Figures 5D, E**) reveals its capacity to selectively amplify reproductive sinks, contrasting with annual crops where wide rows typically boost stem biomass (Peng et al., 2012). Such targeted partitioning was paralleled in nutrient trajectories-T5M’s 17.91% elevation in leaf nitrogen uptake (2021, **Figure 6D**) coincided with 12.60% and 10.54% increases in fruit phosphorus and potassium assimilation (**Figures 6K, L**), indicating row spacing differentially regulates macronutrient translocation pathways. This aligns with potassium’s established role in osmotic regulation during banana fruit filling (Tang et al., 2025), while the temporal progression from nitrogen-dominated (2020) to balanced NPK enhancement (2021) suggests developmental-stage-dependent nutrient remobilization. The 14.71% fruit potassium surplus in T5M versus T3M (**Figure 6L**) may further contribute to lignin deposition against *Fusarium* invasion-a potential synergy between spacing-induced nutrition and pathogen resistance warranting molecular verification. These findings collectively demonstrate how strategic spatial arrangements can precisely modulate dry matter allocation and nutrient partitioning dynamics in perennial cropping systems.

### 4.3 Soil microbiome restructuring and soil fertility

The implementation of ultra-wide-narrow row planting configurations has yielded profound impacts on soil biogeochemical processes while maintaining fundamental edaphic properties (**Figures 7A, B**). Notably, the 5-meter spacing (T5M) enhanced soil AN by 12.33-18.19% alongside significant AK improvements (+11.46% versus T6M) (**Figures 7C, E**), demonstrating distinct nutrient modulation capabilities compared to traditional 3-meter spacing’s phosphorus-focused optimization (Gao et al., 2025). Changes in environmental conditions can alter microbial community structures, thereby affecting soil fertility (Chen et al., 2017; Wang et al., 2018). Soil microbial assemblage responses to ultra-wide-narrow row configurations reveal previously underestimated ecological dimensions of planting architecture design. The treatment-specific divergence in fungal diversity indices (13.51% higher Simpson index in T5M vs T6M, **Figure 8H**) contrasted with bacterial community stability, reflecting fungi’s heightened sensitivity to rhizosphere microhabitat alterations (Deng et al., 2019; Zhou et al., 2024). T5M’s dual enhancement of Actinobacteriota (26.03% vs T3M) and Basidiomycota (136.04% vs T3M) (**Figure 9A**) while suppressing *Fusarium* (31.50% reduction, **Figure 9C**) establishes a novel biological control paradigm-Actinobacteriota-driven chitinolysis likely synergizes with Basidiomycota’s competitive exclusion to create antagonistic networks against soil-borne pathogens (Hui et al., 2020; Djemouai et al., 2023; Lee et al., 2023). Redundancy analysis identified organic matter as the primary driver of microbial structuring (**Figures 9G–I**), with AP emerging as a key correlate of *Fusarium* prevalence (**Figure 9F**). Regarding the suppression of *Fusarium*, the correlation analysis revealed significant negative associations between AP and *Fusarium* abundance (r=-0.72, p<0.01), suggesting AP’s dominant role in pathogen suppression. Although the correlation was not statistically significant (r=-0.52, p=0.17), Actinobacteria enrichment suggests potential microbial involvement that merits consideration alongside chemical factors in future investigations. Furthermore, under a uniform fertilization regime, this study presents the first comprehensive assessment of ultra-wide-narrow row configurations in banana cultivation systems, analyzing their effects on pedochemical profiles and microbiome assemblages. However, the temporal and methodological constraints inherent to this investigation necessitate further research to fully elucidate the causal relationships between planting geometry and soil biotic and abiotic interactions.

### 4.4 Toward sustainable banana production systems

The 5 m ultra-wide-narrow row system operationalizes ecological intensification principles by reconciling competing agricultural objectives. Its capacity to stabilize crop yields, enhance nutrient-use efficiency, and shape disease-suppressive soil microbiomes demonstrates significant transformative potential for perennial cropping systems. This study uses a systems agronomy approach to evaluate planting configurations as integrated units, acknowledging that crop performance arises from spatial synergies. Factorial experiments are essential to clarify the distinct roles of interplant spacing and spatial arrangement in observed improvements. Its mechanization compatibility addresses critical labor constraints while its spatial configuration creates opportunities for integrated pest management through biological control pathways. However, the observed phosphorus trade-offs between T3M and T5M configurations suggest potential synergies with organic amendments or mycorrhizal inoculants. Future research directions should elucidate root exudate-mediated microbial signaling and evaluate longitudinal soil health trajectories under these spatial regimes. The integration of intercropping with crops such as corn, peanuts, or medicinal plants could further enhance land and light utilization, increase beneficial soil microorganisms, and unlock additional production and efficiency potential. This study establishes ultra-wide-narrow row planting as a scalable framework that transcends conventional yield-ecology trade-offs in banana production systems.

## 5 Conclusion

The study establishes ultra-wide-narrow row planting (T5M) as a transformative agronomic strategy unifying productivity enhancement with ecological resilience in banana systems. T5M maintained yield parity with conventional spacing while optimizing sink-driven nutrient allocation and suppressing *Fusarium* through Actinobacteriota-mediated soil microbiome modulation. The configuration preserved microbial diversity but restructured fungal assemblages to favor biocontrol taxa, demonstrating spatial optimization as a lever for integrated disease management. These findings illustrate how spatial rearrangement aligns agronomic objectives with ecological management, offering a sustainable alternative to traditional monoculture practices. Mechanization compatibility reinforces its practical feasibility, warranting broader adoption in banana production systems. Future metabolomic investigations are warranted to unravel root exudate-microbe interactions underlying these spatial benefits, advancing precision agriculture for banana systems.

## Data Availability

The original contributions presented in the study are publicly available. This data can be found here: NCBI BioProject PRJNA1416626.
