# Supplementary figures and images for: Enhanced banana productivity, soil microbial community structure, and Fusarium wilt resistance by ultra-wide-narrow row planting pattern
Source: Front Plant Sci. 2026 Feb 25;16:1753867. doi: 10.3389/fpls.2025.1753867 (PMC12975992; doi:10.3389/fpls.2025.1753867)

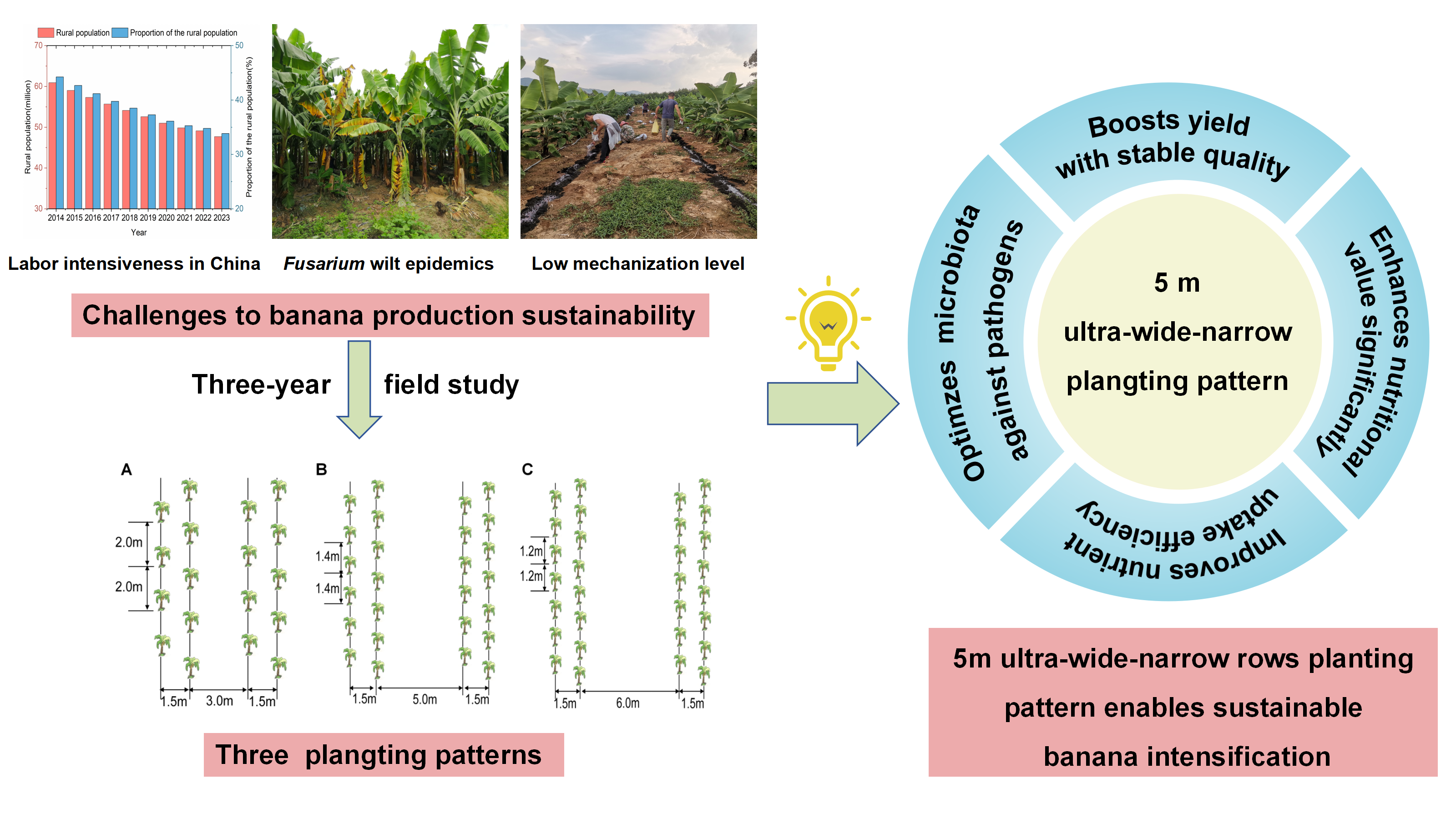

Supplement: Supplementary file 2 [file Image1.tif]
